# Age‐related changes in mean corpuscular volumes in patients without anaemia: An analysis of large‐volume data from a single institute

**DOI:** 10.1111/jcmm.17397

**Published:** 2022-05-22

**Authors:** Jin Young Lee, Hanlim Choi, Jin Woo Park, Bo Ra Son, Jong Hyock Park, Lee Chan Jang, Jae Gil Lee

**Affiliations:** ^1^ Deparment of Trauma Surgery, Trauma Center Chungbuk National University Hospital Cheongju Korea; ^2^ Department of Surgery Chungbuk National University Hospital Cheongju Korea; ^3^ Department of Surgery, College of Medicine Chungbuk National University Cheongju Korea; ^4^ Department of Laboratory Medicine, College of Medicine Chungbuk National University Cheongju Korea; ^5^ College of Medicine Chungbuk National University Cheongju Korea; ^6^ Institute of Health & Science Convergence Chungbuk National University Cheongju Korea; ^7^ Department of Surgery Yonsei University College of Medicine Seoul Korea

**Keywords:** age, complete blood count, mean corpuscular volume

## Abstract

Although the mean corpuscular volume (MCV) has been associated with various diseases, these associations in relation to the age‐related trends in MCV remain unclear. Therefore, we used a dataset with over one million values to identify the relationship between ageing and MCV changes. All laboratory data obtained between November 1998 and November 2019 at Chungbuk National University Hospital were retrospectively collected. After excluding cases with missing values for individual complete blood count parameters, outlier MCV values, and ages less than 1 year and more than 88 years, 977,335 MCV values were obtained from 309,393 patients. Principal component analysis of blood components with ages and analysis of the median value changes for each blood component across decade‐wise age groups were conducted to identify relationships between ageing and changes in blood components. The median values of MCV showed gradual increments with age. The linear relationship for patients aged 1–25 years had a larger slope than that for patients aged 26–88 years. For MCV, the equation for patients aged 1–25 years was 0.40*(age) + 81.24 in females and 0.45*(age) + 79.58 in males. The equation for patients aged 26–90 years was 0.04*(age) + 88.97 in females and 0.06*age + 88.30 in males. Among patients aged >40 years, the MCV value was higher in men than in women. Analysis of a large dataset showed that the MCV gradually increased with age and the linear relationship differed between patients aged 1–25 and 26–88 years.

## 1 INTRODUCTION

Complete blood count (CBC) tests, which are commonly used during health assessments, can provide information regarding anaemia, infection or impaired haemostasis.^1^ CBCs usually include measurements of the white blood cell (WBC) count, WBC differential count, red blood cell (RBC) count, haematocrit (Ht; the volume of percentage of RBCs), haemoglobin (Hb) levels, mean corpuscular volume (MCV), mean corpuscular haemoglobin level, mean corpuscular haemoglobin concentration, red cell distribution width (RDW) and platelet count.^2^

A diagnosis of anaemia is based on haemoglobin levels less than 13 g/dl in males and 12 g/dl in females,^3^ and the MCV value can be used to classify anaemia as either microcytic, normocytic or macrocytic.^4^, ^5^ However, MCV measurements are inevitably less useful in patients without anaemia. One study evaluating the clinical importance of an elevated MCV value irrespective of the haemoglobin value revealed that such elevated values were commonly associated with alcoholism and hepatic disease, malignant disease, and the effects of chemotherapy.^6^ More recently, MCV has been suggested to be a prognostic factor for colorectal or oesophageal cancer,^7^, ^8^ chronic kidney disease,^9^ and ischaemic stroke or coronary interventions.^10^, ^11^ However, these studies did not attempt to evaluate their findings in the context of the age‐related trends in MCV that have been described in several reports.^12^, ^13^, ^14^ Moreover, these studies had a relatively small sample size and did not include regression analyses.

In a pilot study using health‐check data from May 2001 to November 2019 for patients aged 11–95 years and including 66,911 values each of MCV, Hb, haematocrit (Hct) and RBC,^15^ the MCV showed the most distinct age‐related trends. Therefore, we conducted this study using the CBC data obtained over a 21‐year period at our hospital to determine a regression model for ageing and MCV changes.

## 2 MATERIALS AND METHODS

### 2.1 Study population and design

All laboratory data obtained between November 1998 and November 2019 from health checks as well as inpatients, outpatients, and emergency room patients aged 1–99 years at our hospital were collected for this study. The data were anonymized and handled confidentially. The total laboratory data of 502,978 patients were collected, and 328,752 patients without anaemia were enrolled. The diagnosis of anaemia in males and females is based on threshold haemoglobin levels of 13 and 12 g/dl, respectively. Data for CBC parameters such as RBC count, Hct values, Hb levels, and MCV and RDW values were collected, yielding 1,036,616 values for individual CBC parameters. We excluded 3506 missing values for individual CBC parameters, 38,431 outlier MCV, RDW values and 17,344 values obtained from patients aged less than 1 year and more than 88 years because of less than 1000 observations in each age group. Outliers were defined as values over or under the 3/4 quantile + (1.51* inter‐quantile range) and 1/4 quantile–(1.51*inter‐quantile range). Finally, 977,335 values for individual CBC parameters from 309,393 patients aged 1–88 years were included. This study was approved by the Institutional Review Board of Chungbuk University Hospital (2021–10‐023). The review board waived the requirement for informed consent due to the retrospective nature of the study.

### 2.2 Data collection sequence

All patients who visited the hospital were recorded in the master table, and all hospital data were obtained from this master table. The laboratory table, which included information regarding age, sex, order‐date, laboratory (lab) test date, laboratory values and laboratory test names, was obtained by querying the client and server system. Queries were made for text files, which were transformed using MySQL Workbench software version 8.0 database (Oracle Corporation).

The number of patients who visited the hospital and received a doctors' order was 811,050, and 502,978 patients underwent laboratory tests. Data for CBC parameters, such as RBC count, Hct values, Hb levels, and MCV and RDW values, were collected. We included WBC, WBC count, PLT and PLT count in these searches, but did not include the corresponding data in this study.

### 2.3 Statistical analysis

Statistical analyses were performed using R software version 4.1.0 (The R Foundation). The normality of the data was checked using the Anderson–Darling test. Principal component analysis (PCA) was used to evaluate bivariate cross‐correlations among CBC parameters and age. The Kruskal–Wallis test and post hoc evaluations by the Tukey method were performed to identify the statistical differences in CBC parameters in decade‐wise age groups. Comparisons between sexes were performed using the Mann–Whitney *U*‐test. Results were considered to be statistically significant when the *p*‐value was less than 0.05. A linear regression analysis using the least‐squares method was performed to evaluate the changes in median MCV value in every 1 year.

### 2.4 Definitions of CBC parameters and anaemia

1. Hb level is the concentration of haemoglobin in whole blood, in grams/decilitre (g/dl).
2. RBC count is the number of RBCs per microliter of blood (or number of RBCs * 10^6^/μl).
3. Hct is the percentage of blood that is represented by the red blood cells.
4. MCV is the average size and volume of red blood cells. It can be calculated by multiplying the per cent haematocrit by 10 divided by the erythrocyte count.
5. The RDW is calculated using the formula RDW = (standard deviation of MCV ÷ MCV) × 100^16^.
6. Anaemia was defined by a haemoglobin level less than 13 g/dl in males and 12 g/dl in females.^3^

## 3 RESULTS

### 3.1 Characteristics of the MCV data

**FIGURE 1:**
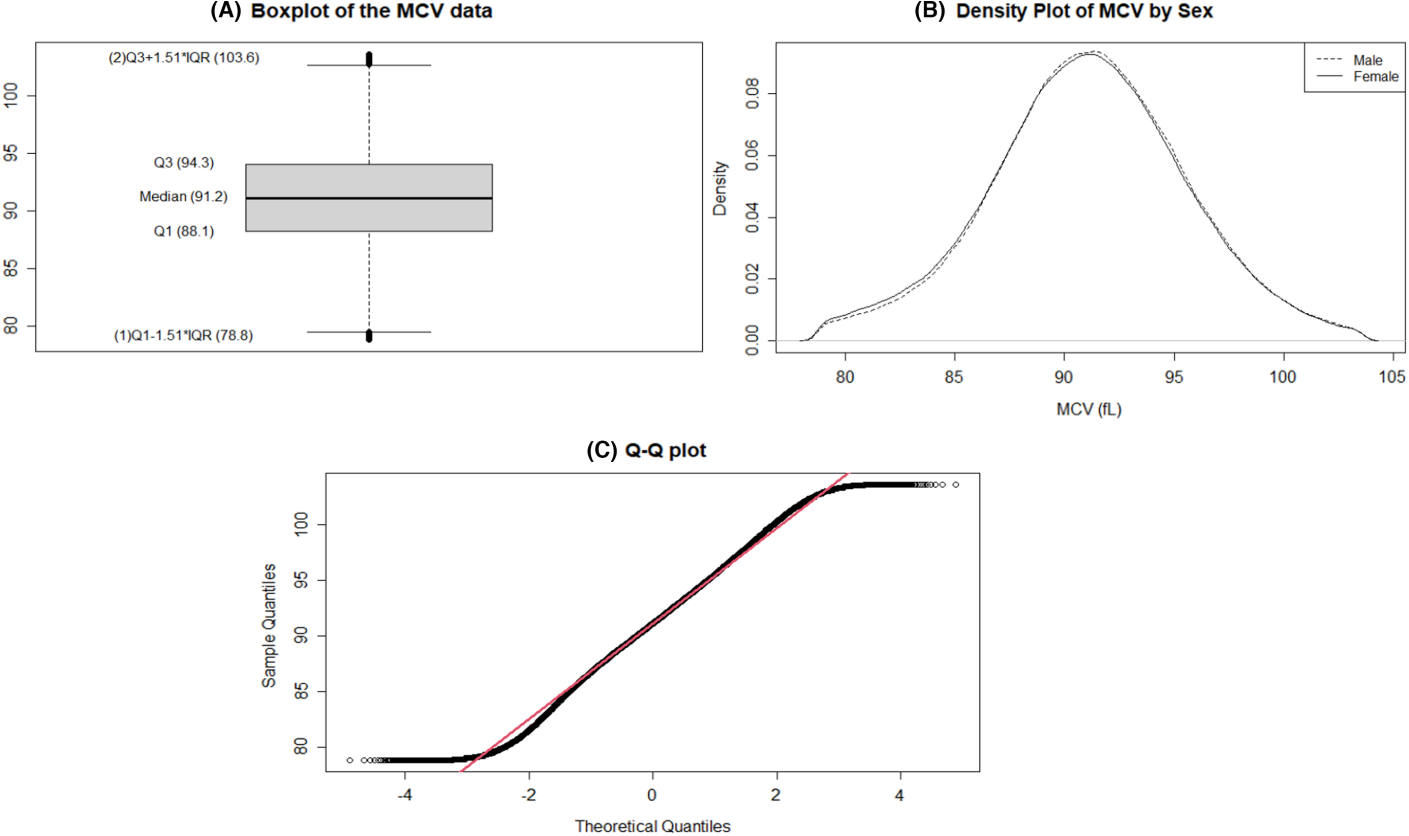
Characteristics of MCV data. (A) Boxplot of the MCV data after outlier removal. (B) Density plot of MCV data. (C) Q‐Q plot of MCV data

A boxplot and density plot were generated to inspect the entire MCV data after outlier removal. The median MCV value was 91.2 fl, and the lower quartile (Q1) and upper quartile (Q3) values were 88.1 and 94.3 fl, respectively (Figure 1A). The distribution of MCV values was similar for male and female patients (Figure 1B). However, the data did not pass the normality test for statistical analyses using the quantile–quantile (Q‐Q) plot, which showed a bimodal distribution, and the Anderson–Darling normality test (*p* < 0.05).

### 3.2 Principal component analysis of all ages in the entire data

**FIGURE 2:**
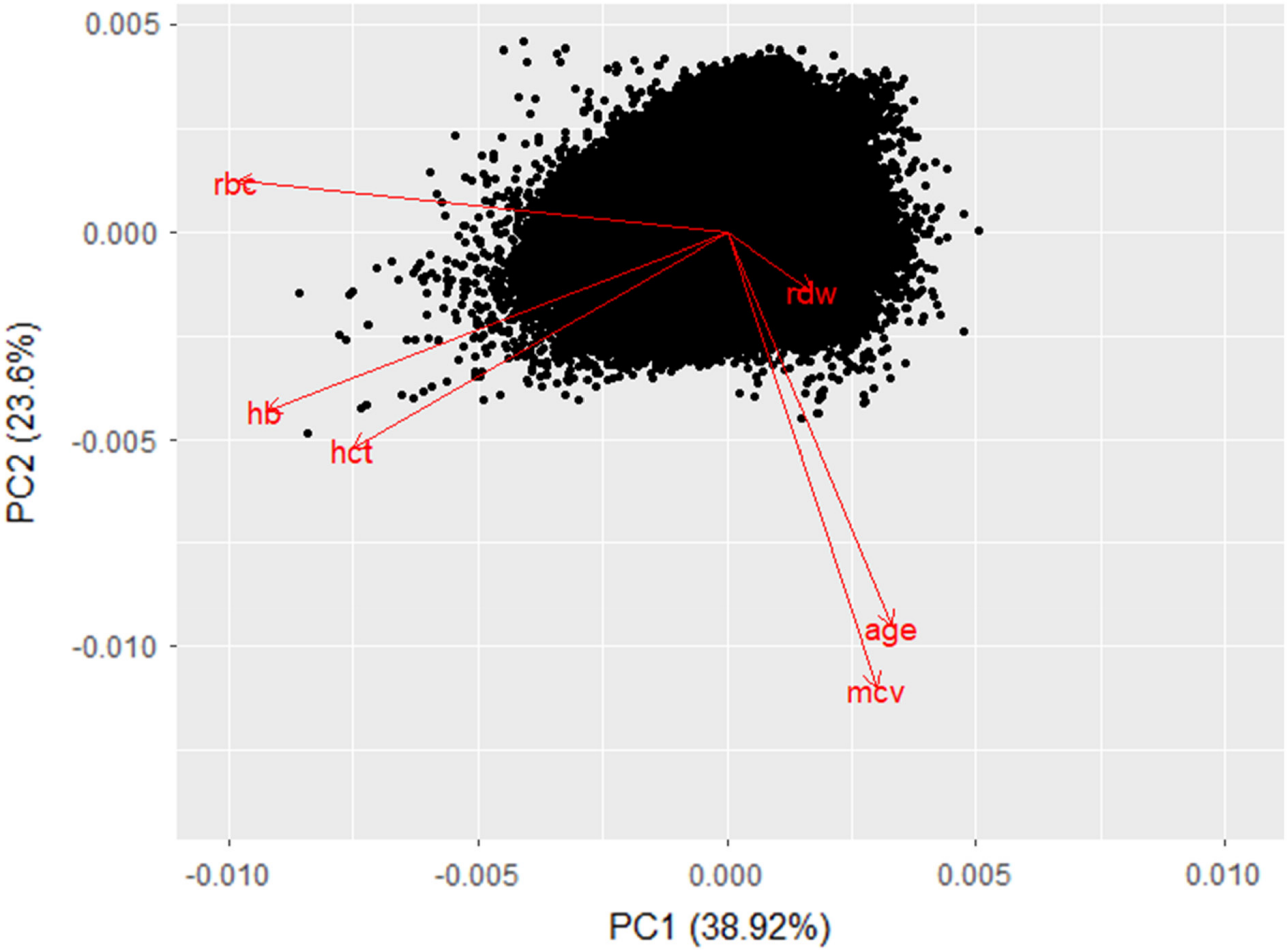
Biplot of principal component analysis of blood components with ages

Principal component analysis (PCA) was performed to strengthen the hypothesis of a correlation between MCV and ageing compared with other CBC parameters. The cumulative proportion of principal component 2 was 61.25% (Table S1). The biplot of PCA showed that the correlation between age and the MCV followed the same direction as that for the other parameters (Figure 2).

### 3.3 Changes in MCV and other CBC components in decade‐wise age groups

We divided the patients into nine decade‐wise age groups. The median MCV values gradually increased in the age groups. The Kruskal–Wallis test was conducted to identify statistical differences in age groups. All CBC parameters showed significant differences according to the age groups; however, the median values of Hb, Hct, RBC and RDW showed no changes up to the first decimal place (Table 1). The post hoc test with the Tukey method showed all age groups were significant differences in MCV. Finally, the MCV median values gradually increased with age, with significant differences among age groups.

**TABLE 1:** The changes in MCV and other CBC components in decade‐wise age groups

Age group (years)	1–9	10–19	20–29	30–39	40–49	50–59	60–69	87–79	80–88	*p*‐value^a^
Count, *n*	27,793	48,305	94,281	111,288	156,606	207,670	176,107	122,709	32,576	
MCV, fl (median, IQR)	82.2 (80.5–84.3)	87.4 (84.7–90.1)	89.9 (87.4–92.5)	90.4 (87.7–93.1)	91.2 (88.5–93.9)	91.5 (88.9–94.3)	92.1 (89.4–94.9)	92.6 (89.8–95.4)	93.0 (90.2–95.9)	<0.001^b^
Hb, g/dl (median, IQR)	13.2 (12.7–13.6)	14.0 (13.2–15.0)	14.0 (13.2–15.0)	14.0 (13.2–15.0)	14.0 (13.2–15.0)	14.0 (13.2–15.0)	14.0 (13.2–15.0)	14.0 (13.2–15.0)	14.0 (13.2–15.0)	<0.001
Hct, % (median, IQR)	38.0 (35.8–39.7)	38.0 (35.8–39.7)	38.0 (35.8–39.7)	38.0 (35.8–39.7)	38.0 (35.8–39.7)	38.0 (35.8–39.7)	38.0 (35.8–39.7)	38.0 (35.8–39.7)	38.0 (35.8–39.7)	<0.001
RBC, g/dl (median, IQR)	4.7 (4.5–4.9)	4.7 (4.5–4.9)	4.7 (4.5–4.9)	4.7 (4.5–4.9)	4.7 (4.5–4.9)	4.7 (4.5–4.9)	4.7 (4.5–4.9)	4.7 (4.5–4.9)	4.7 (4.5–4.9)	<0.001
RDW, % (median, IQR)	12.0 (12.0–13.0)	12.0 (12.0–13.0)	12.0 (12.0–13.0)	12.0 (12.0–13.0)	12.0 (12.0–13.0)	12.0 (12.0–13.0)	12.0 (12.0–13.0)	12.0 (12.0–13.0)	12.0 (12.0–13.0)	<0.001

Abbreviations: CBC, Complete blood count; Hb, haemoglobin; Hct, haematocrit; IQR, interquartile range; MCV, mean corpuscular volume; RBC, red blood cell; RDW, red cell distribution width.

*p*‐value from the Kruskal–Wallis test.

All age groups showed significant differences in post hoc tests with the Tukey method.

**FIGURE 3:**
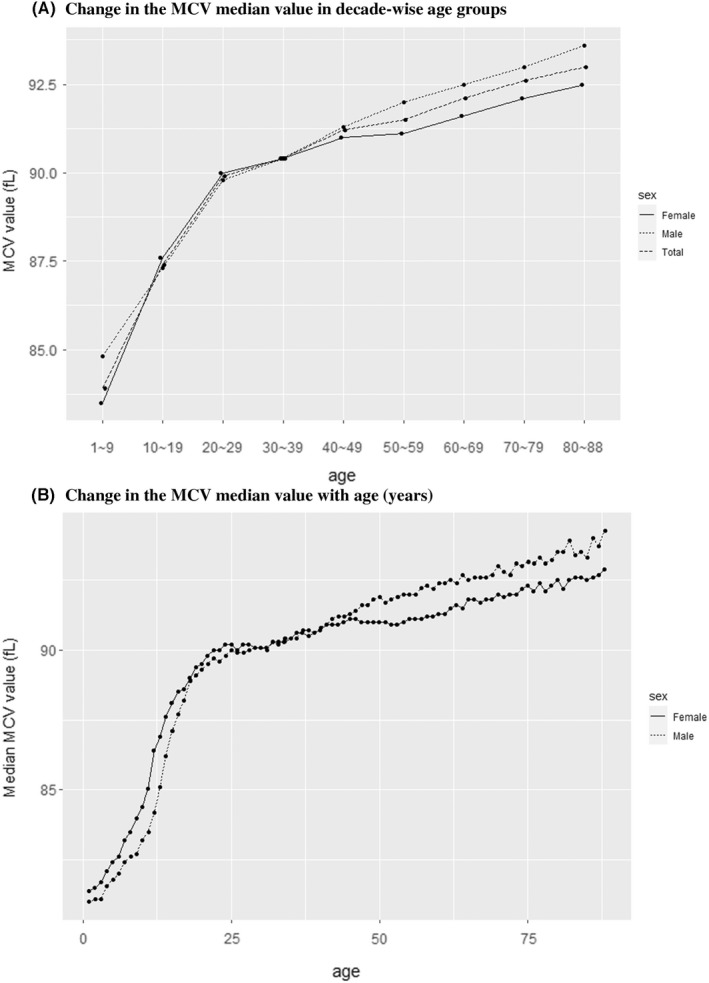
(A) Changes in the MCV median value in decade‐wise age groups. (B) Changes in the MCV median value with age (years)

The MCV among females was significantly higher until the third decade than among males. However, after the fourth decade, the MCV of males was significantly higher than that of females (Table 2). Figure 3 illustrates the decade‐wise age‐dependent increase in the MCV in both sexes.

**TABLE 2:** The changes in MCV values in both sexes categorized according to decade‐wise age groups

Age group (years)	Count (*n*)	MCV value, fl (median, IQR)			*p*‐value
		Total	Female	Male	
1–9	27,793	82.2 (80.5–84.3)	82.4 (80.7–84.5)	81.9 (80.3–83.9)	<0.001
10–19	48,305	87.4 (84.7–90.1)	87.6 (84.9–90.3)	87.3 (84.6–90.0)	<0.001
20–29	94,281	89.9 (87.4–92.5)	90.0 (87.5–92.6)	89.8 (87.4–92.3)	<0.001
30–39	111,288	90.4 (87.7–93.1)	90.4 (87.6–93.2)	90.4 (87.9–93.1)	<0.001
40–49	156,606	91.2 (88.5–93.9)	91.0 (88.2–93.7)	91.3 (88.7–94.1)	<0.001
50–59	207,670	91.5 (88.9–94.3)	91.1 (88.5–93.7)	92 (89.3–94.8)	<0.001
60–69	176,107	92.1 (89.4–94.9)	91.6 (89.0–94.3)	92.5 (89.8)–95.3)	<0.001
70–79	122,709	92.6 (89.8–95.4)	92.1 (89.4–94.9)	93 (90.2–95.9)	<0.001
80–88	32,576	93.0 (90.2–95.9)	92.5 (89.7–95.4)	93.6 (90.8–96.5)	<0.001

*Note: p*‐value from Mann–Whitney *U*‐test.

Abbreviations: IQR, Interquartile range; MCV, mean corpuscular volume.

### 3.4 Linear regression analysis of MCV and age

**FIGURE 4:**
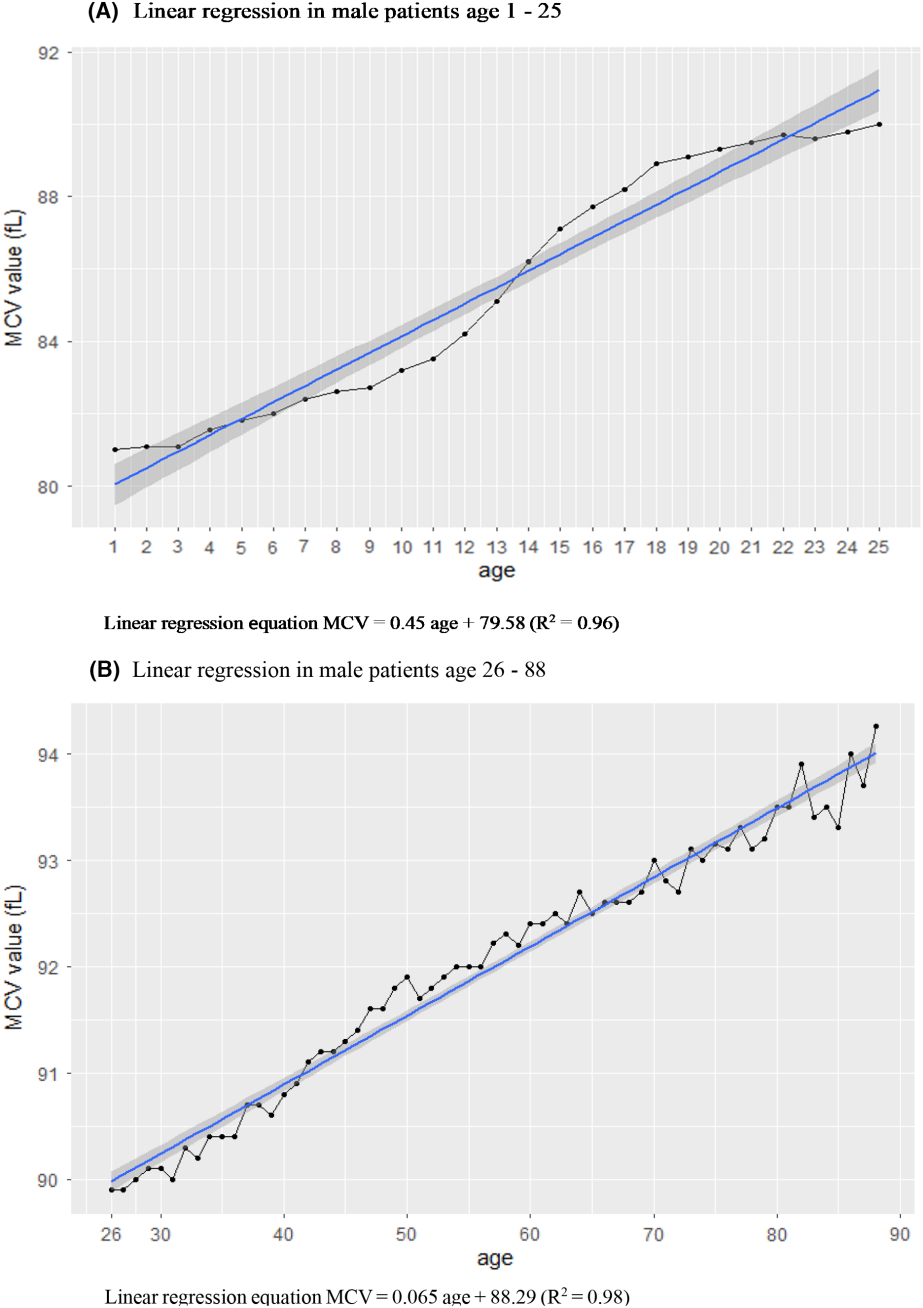
(A) Linear regression in male patients aged 1–25 years. (B) Linear regression in male patients aged 26–88 years

**FIGURE 5:**
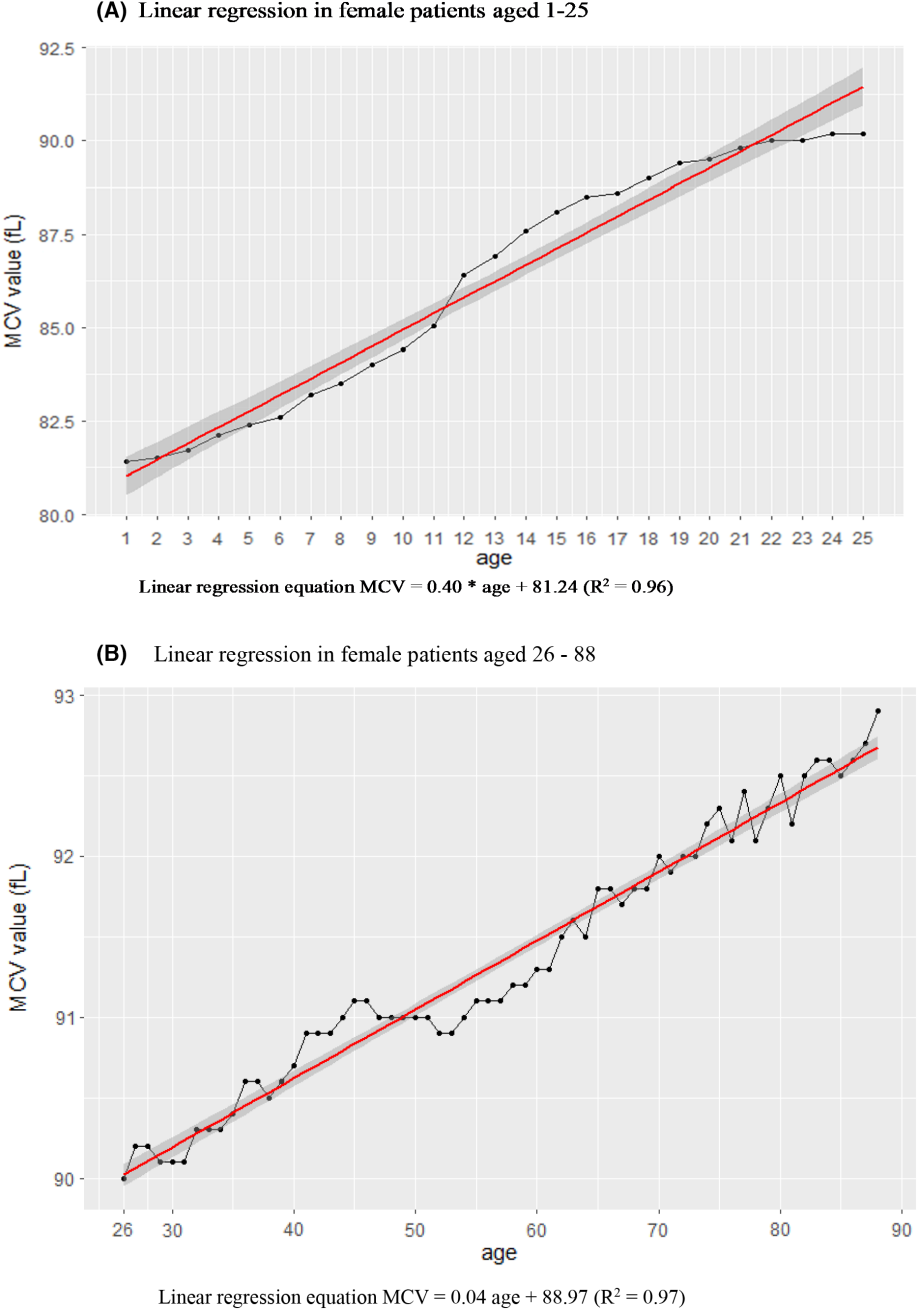
(A) Linear regression in female patients aged 1–25 years. (B) Linear regression in female patients aged 26–88 years

The age‐wise changes in the median MCV value are shown in Figure 3B and the Table S2. We obtained two linear regression equations for the relationship of MCV with age. The linear relationship for patients aged 1–25 years had a larger slope than that for patients aged 26–88 years. The equation for patients aged 1–25 years was MCV = 0.40*(age) + 81.24 (*R* ^2^ = 0.97) in females and 0.45*(age) + 79.58 (*R* ^2^ = 0.98) in males. Among patients aged 26–90 years, the equation was MCV = 0.04*(age) + 88.97 (*R* ^2^ = 0.96) in females and 0.06*age + 88.30 (*R* ^2^ = 0.98) in males (Figures 4 and 5).

## 4 DISCUSSION

This study included more patient data than any of the previous studies,^12^, ^13^, ^14^, ^17^, ^18^ and the findings revealed a distinct age‐related increase in MCV compared with other CBC parameters. The MCV increased gradually from 1 to 88 years of age, and the increase could be represented by two different sets of linear regression Equations.

MCV is used to classify anaemia as either microcytic, normocytic or macrocytic.^5^ In microcytic anaemia, the average erythrocyte volume is smaller than normal, generally under 80 fl, and this finding is commonly observed in cases of chronic iron‐deficient anaemia, anaemia of chronic disease and thalassemias. Macrocytic anaemia is characterized by elevated MCV (generally over 100 fl) and is subcategorized as megaloblastic or non‐megaloblastic. Megaloblastic anaemia is caused by impaired DNA synthesis and commonly occurs in folate deficiency and vitamin B12 deficiency, while non‐megaloblastic anaemia occurs due to hepatic insufficiency, chronic alcoholism and rare congenital diseases.^5^

The MCV has also been recently reported to be a risk factor for mortality and morbidity in some clinical settings. Borderline high MCV (MCV > 95 fl) was independently associated with arterial stiffness measured by brachial‐ankle pulse wave velocity in apparently healthy individuals.^19^ In patients with stage 3–5 chronic kidney disease (CKD), MCV was associated with all‐cause mortality, cardiovascular mortality and infection‐associated mortality.^9^ Other studies with CKD patients showed that an increase in MCV was independently associated with more impaired endothelial function and insulin resistance and showed higher cardiovascular event and mortality rates.^20^ In older patients, high MCV levels were also significantly associated with high rates of decline on tasks of global mental status, long‐delay memory and attention, even after adjusting for potential confounders.^21^

Age‐related changes in MCV may be a part of the normal ageing process. Young RBCs are released into circulation as reticulocytes from the bone marrow, and old RBCs will be removed from circulation within a few hours or days.^22^ The lifespan of RBCs in older individuals is shorter than that in young adults, and the RBC production increases to compensate for the shorter lifespan.^14^, ^23^ These younger RBCs tend to have larger volumes, as measured by MCV.^14^ Various cytokines and growth factors are involved in the formation of RBCs, including erythropoietin, which is essential for the survival and differentiation of erythropoietic progenitors.^24^, ^25^ Other growth factors and cytokines, such as insulin, insulin‐like growth factors (IGFs), hepatocyte growth factors and interleukins (IL‐3, IL‐4, IL‐6, IL‐9 and IL‐11), have also been known to co‐stimulate of growth of the RBCs.^24^, ^26^ The relationship between biological changes and ageing is not fully understood. One recent study revealed that prolonged growth hormone, insulin and IGF nutrient response signalling pathways were silent killers of stem cells and one the factors responsible for ageing.^27^ Ageing accelerates after reproductive age, and several mechanisms are known to accelerate this process.^28^ The age‐related changes in MCV showed two linear trends with different slopes, with the distinction appearing from 25 years of age. This result may provide evidence for accelerated ageing after reproductive age, but more biological studies are needed to validate this assumption. Moreover, additional research is required to explain the link among IGF and MCV.

Our study had several limitations. First, the retrospectively obtained patient cohort included inpatients, outpatients and emergency room patients in addition to those who had undergone medical check‐ups. Therefore, these patients were more likely to have disease and may not represent the general population. Second, we could not control the influence of factors other than anaemia, including underlying diseases such as chronic hepatitis, chronic renal disease or malignancy disease, on MCV changes, and the influence of these factors should be verified in future studies. Third, we did not include most important clinical outcome, that is mortality. Further studies are needed to identify the relation between an increase in MCV with ageing and mortality.

## 5 CONCLUSIONS

Cleaning of medical data involves substantial data loss. Our analysis of a large data sample from Chungbuk National University Hospital showed that the MCV gradually increased with age. The MCV values showed two distinct linear relationships in patients aged 1–25 and 26–88 years. Female patients aged 40–80 years showed lower MCV values than males patients of the same age group.

## AUTHOR CONTRIBUTIONS

**Jin Young Lee:** Conceptualization (equal); data curation (equal); formal analysis (equal); writing – original draft (equal); writing – review and editing (equal). **Hanlim Choi:** Data curation (equal); validation (equal); writing – original draft (supporting); writing – review and editing (equal). **Jin Woo Park:** Conceptualization (equal); formal analysis (equal); supervision (equal); validation (equal); writing – review and editing (equal). **Bo Ra Son:** Supervision (equal); validation (equal); writing – review and editing (equal). **Jong Hyock Park:** Supervision (equal); validation (equal); writing – review and editing (equal). **Lee Chan Jang:** Conceptualization (lead); data curation (lead); formal analysis (lead); methodology (lead); project administration (lead); supervision (lead); validation (lead); writing – original draft (equal); writing – review and editing (equal). **Jae Gil Lee:** Supervision (equal); writing – review and editing (equal).

## CONFLICT OF INTEREST

The authors declare no competing financial interests.

## Supporting information


Table S1

Table S2

## Data Availability

The datasets generated during and/or analysed during the current study are not publicly available due to our institutional policy.
