# Supplementary material for: Age‐related changes in mean corpuscular volumes in patients without anaemia: An analysis of large‐volume data from a single institute
Source: J Cell Mol Med. 2022 May 22;26(12):3548–56. doi: 10.1111/jcmm.17397 (PMC9189337; doi:10.1111/jcmm.17397)
Supplement: Supplementary file 1 — Table S1 [file JCMM-26-3548-s002.docx]

Supplemental table 1. Importance of components:

|  | PC1 | PC2 | PC3 | PC4 | PC5 | PC6 |
| --- | --- | --- | --- | --- | --- | --- |
| Standard deviation | 1.5279 | 1.1899 | 1.0114 | 0.8111 | 0.70708 | 0.26240 |
| Proportion of Variance | 0.3891 | 0.2360 | 0.1705 | 0.1096 | 0.08333 | 0.01148 |
| Cumulative Proportion | 0.3891 | 0.6251 | 0.7956 | 0.9052 | 0.98852 | 1.00000 |
