# Supplementary material for: Age‐related changes in mean corpuscular volumes in patients without anaemia: An analysis of large‐volume data from a single institute
Source: J Cell Mol Med. 2022 May 22;26(12):3548–56. doi: 10.1111/jcmm.17397 (PMC9189337; doi:10.1111/jcmm.17397)
Supplement: Supplementary file 2 — Table S2 [file JCMM-26-3548-s001.docx]

Supplemental table 2.

| age | Count  (n) | M:F ratio | Male | | | Female | | |
| --- | --- | --- | --- | --- | --- | --- | --- | --- |
|  |  |  | MCV | MCV | MCV | MCV | MCV | MCV |
|  |  |  | (median) | (Q1) | (Q3) | (median) | (Q1) | (Q3) |
| 1 | 3382 | 3:7 | 81 | 79.8 | 82.7 | 81.4 | 80.1 | 83.2 |
| 2 | 2804 | 2:5 | 81.1 | 79.9 | 83.2 | 81.5 | 80.1 | 83.3 |
| 3 | 2732 | 4:9 | 81.1 | 80 | 83.1 | 81.7 | 80.2 | 83.6 |
| 4 | 2867 | 4:9 | 81.6 | 80.2 | 83.3 | 82.1 | 80.5 | 84 |
| 5 | 3240 | 4:7 | 81.8 | 80.3 | 83.6 | 82.4 | 80.7 | 84.4 |
| 6 | 3124 | 3:5 | 82 | 80.5 | 84.1 | 82.6 | 81 | 84.7 |
| 7 | 3137 | 2:3 | 82.4 | 80.6 | 84.4 | 83.2 | 81.2 | 85.2 |
| 8 | 3203 | 2:3 | 82.6 | 80.8 | 84.6 | 83.5 | 81.7 | 85.7 |
| 9 | 3304 | 5:7 | 82.7 | 81.1 | 84.8 | 84 | 82 | 86 |
| 10 | 3381 | 4:5 | 83.2 | 81.3 | 85.2 | 84.4 | 82.3 | 86.7 |
| 11 | 3168 | 8:9 | 83.5 | 81.4 | 85.5 | 85.1 | 82.8 | 87.4 |
| 12 | 3349 | 1:1 | 84.2 | 82 | 86.5 | 86.4 | 84.1 | 88.9 |
| 13 | 3536 | 5:4 | 85.1 | 83 | 87.6 | 86.9 | 84.5 | 89.2 |
| 14 | 3883 | 4:3 | 86.2 | 83.8 | 88.6 | 87.6 | 85.2 | 90.1 |
| 15 | 4451 | 14:9 | 87.1 | 84.7 | 89.6 | 88.1 | 85.9 | 90.4 |
| 16 | 5239 | 8:5 | 87.7 | 85.5 | 90.1 | 88.5 | 85.9 | 90.8 |
| 17 | 5646 | 12:7 | 88.2 | 86 | 90.5 | 88.6 | 86 | 91.1 |
| 18 | 7046 | 17:9 | 88.9 | 86.5 | 91.2 | 89 | 86.4 | 91.6 |
| 19 | 8606 | 2:1 | 89.1 | 86.8 | 91.6 | 89.4 | 86.8 | 92 |
| 20 | 8165 | 10:7 | 89.3 | 86.9 | 91.7 | 89.5 | 86.9 | 92 |
| 21 | 8147 | 1:1 | 89.5 | 87.3 | 92 | 89.8 | 87.3 | 92.2 |
| 22 | 9462 | 9:8 | 89.7 | 87.4 | 92 | 90 | 87.4 | 92.5 |
| 23 | 9887 | 9:8 | 89.6 | 87.3 | 92.2 | 90 | 87.4 | 92.6 |
| 24 | 9693 | 10:9 | 89.8 | 87.4 | 92.3 | 90.2 | 87.6 | 92.8 |
| 25 | 9872 | 10:9 | 90 | 87.7 | 92.6 | 90.2 | 87.5 | 92.8 |
| 26 | 9731 | 1:1 | 89.9 | 87.5 | 92.5 | 90 | 87.6 | 92.6 |
| 27 | 9722 | 1:1 | 89.9 | 87.5 | 92.5 | 90.2 | 87.6 | 92.7 |
| 28 | 9649 | 1:1 | 90 | 87.5 | 92.6 | 90.2 | 87.6 | 92.8 |
| 29 | 9955 | 1:1 | 90.1 | 87.6 | 92.5 | 90.1 | 87.6 | 92.9 |
| 30 | 10136 | 1:1 | 90.1 | 87.6 | 92.6 | 90.1 | 87.3 | 92.8 |
| 31 | 10383 | 1:1 | 90 | 87.6 | 92.6 | 90.1 | 87.4 | 92.8 |
| 32 | 10590 | 1:1 | 90.3 | 87.7 | 92.9 | 90.3 | 87.6 | 93 |
| 33 | 10800 | 1:1 | 90.2 | 87.6 | 93 | 90.3 | 87.6 | 93.1 |
| 34 | 11177 | 1:1 | 90.4 | 87.9 | 93 | 90.3 | 87.6 | 93.2 |
| 35 | 11125 | 1:1 | 90.4 | 87.9 | 93.1 | 90.4 | 87.7 | 93.3 |
| 36 | 11523 | 7:6 | 90.4 | 88 | 93.2 | 90.6 | 87.7 | 93.4 |
| 37 | 11509 | 7:6 | 90.7 | 88.1 | 93.4 | 90.6 | 87.7 | 93.2 |
| 38 | 11863 | 10:9 | 90.7 | 88.1 | 93.4 | 90.5 | 87.6 | 93.4 |
| 39 | 12182 | 7:6 | 90.6 | 88.1 | 93.3 | 90.6 | 87.8 | 93.5 |
| 40 | 13006 | 6:5 | 90.8 | 88.2 | 93.5 | 90.7 | 87.9 | 93.5 |
| 41 | 13249 | 10:9 | 90.9 | 88.3 | 93.8 | 90.9 | 88.1 | 93.6 |
| 42 | 13739 | 8:7 | 91.1 | 88.6 | 93.8 | 90.9 | 88.1 | 93.7 |
| 43 | 14290 | 8:7 | 91.2 | 88.6 | 93.9 | 90.9 | 88 | 93.6 |
| 44 | 15480 | 9:8 | 91.2 | 88.6 | 94 | 91 | 88.2 | 93.7 |
| 45 | 15882 | 9:8 | 91.3 | 88.7 | 94 | 91.1 | 88.3 | 93.7 |
| 46 | 17225 | 9:8 | 91.4 | 88.8 | 94.2 | 91.1 | 88.4 | 93.8 |
| 47 | 17532 | 9:8 | 91.6 | 89 | 94.3 | 91 | 88.2 | 93.7 |
| 48 | 18408 | 1:1 | 91.6 | 88.9 | 94.4 | 91 | 88.3 | 93.8 |
| 49 | 17798 | 1:1 | 91.8 | 89.2 | 94.5 | 91 | 88.3 | 93.7 |
| 50 | 19635 | 1:1 | 91.9 | 89.1 | 94.7 | 91 | 88.4 | 93.6 |
| 51 | 19864 | 1:1 | 91.7 | 89.1 | 94.5 | 91 | 88.4 | 93.6 |
| 52 | 20589 | 1:1 | 91.8 | 89.2 | 94.5 | 90.9 | 88.3 | 93.6 |
| 53 | 21139 | 1:1 | 91.9 | 89.2 | 94.7 | 90.9 | 88.4 | 93.5 |
| 54 | 21175 | 1:1 | 92 | 89.4 | 94.8 | 91 | 88.4 | 93.7 |
| 55 | 21092 | 1:1 | 92 | 89.3 | 94.8 | 91.1 | 88.4 | 93.8 |
| 56 | 22118 | 1:1 | 92 | 89.4 | 94.9 | 91.1 | 88.6 | 93.8 |
| 57 | 21025 | 10:9 | 92.2 | 89.5 | 95.1 | 91.1 | 88.6 | 93.9 |
| 58 | 21148 | 9:8 | 92.3 | 89.6 | 95.2 | 91.2 | 88.6 | 93.8 |
| 59 | 19885 | 7:6 | 92.2 | 89.5 | 95 | 91.2 | 88.7 | 94 |
| 60 | 20270 | 7:6 | 92.4 | 89.6 | 95.1 | 91.3 | 88.7 | 94 |
| 61 | 19120 | 6:5 | 92.4 | 89.7 | 95.3 | 91.3 | 88.8 | 94 |
| 62 | 19339 | 7:6 | 92.5 | 89.7 | 95.3 | 91.5 | 88.9 | 94.1 |
| 63 | 18425 | 6:5 | 92.4 | 89.6 | 95.2 | 91.6 | 89 | 94.3 |
| 64 | 17820 | 6:5 | 92.7 | 90 | 95.4 | 91.5 | 88.8 | 94.2 |
| 65 | 17111 | 9:8 | 92.5 | 89.7 | 95.4 | 91.8 | 89 | 94.5 |
| 66 | 16830 | 7:6 | 92.6 | 89.8 | 95.4 | 91.8 | 89.1 | 94.5 |
| 67 | 15964 | 9:8 | 92.6 | 89.9 | 95.4 | 91.7 | 89.2 | 94.6 |
| 68 | 16187 | 9:8 | 92.6 | 89.9 | 95.4 | 91.8 | 89.2 | 94.6 |
| 69 | 15041 | 1:1 | 92.7 | 89.9 | 95.5 | 91.8 | 89.2 | 94.6 |
| 70 | 15009 | 1:1 | 93 | 90.1 | 95.9 | 92 | 89.2 | 94.8 |
| 71 | 14435 | 9:8 | 92.8 | 90 | 95.8 | 91.9 | 89.2 | 94.6 |
| 72 | 14151 | 1:1 | 92.7 | 90 | 95.7 | 92 | 89.3 | 94.7 |
| 73 | 13805 | 1:1 | 93.1 | 90.3 | 95.9 | 92 | 89.3 | 94.7 |
| 74 | 13526 | 1:1 | 93 | 90.3 | 95.8 | 92.2 | 89.6 | 94.9 |
| 75 | 12242 | 1:1 | 93.2 | 90.4 | 95.9 | 92.3 | 89.6 | 95 |
| 76 | 11497 | 1:1 | 93.1 | 90.2 | 96 | 92.1 | 89.3 | 95 |
| 77 | 10583 | 1:1 | 93.3 | 90.5 | 96.5 | 92.4 | 89.8 | 95.3 |
| 78 | 9307 | 1:1 | 93.1 | 90.3 | 96.1 | 92.1 | 89.4 | 94.9 |
| 79 | 8154 | 1:1 | 93.2 | 90.5 | 96.5 | 92.3 | 89.7 | 95.1 |
| 80 | 7582 | 5:6 | 93.5 | 90.8 | 96.4 | 92.5 | 89.8 | 95.6 |
| 81 | 5966 | 5:6 | 93.5 | 90.8 | 96.5 | 92.2 | 89.5 | 95.3 |
| 82 | 4997 | 3:4 | 93.9 | 90.8 | 96.7 | 92.5 | 89.8 | 95.5 |
| 83 | 3961 | 3:4 | 93.4 | 90.7 | 96.4 | 92.6 | 89.9 | 95.4 |
| 84 | 3251 | 5:7 | 93.5 | 90.8 | 96.5 | 92.6 | 89.5 | 95.4 |
| 85 | 2494 | 5:9 | 93.3 | 90.7 | 95.8 | 92.5 | 89.7 | 95.5 |
| 86 | 1802 | 3:5 | 94 | 91.4 | 96.7 | 92.6 | 89.7 | 95.2 |
| 87 | 1326 | 3:5 | 93.7 | 91.3 | 96.2 | 92.7 | 89.3 | 95.5 |
| 88 | 1197 | 3:5 | 94.3 | 91.1 | 97.3 | 92.9 | 89.8 | 95.5 |
